# Rapid Full-Cycle Technique to Control Adulteration of Meat Products: Integration of Accelerated Sample Preparation, Recombinase Polymerase Amplification, and Test-Strip Detection

**DOI:** 10.3390/molecules26226804

**Published:** 2021-11-11

**Authors:** Aleksandr V. Ivanov, Demid S. Popravko, Irina V. Safenkova, Elena A. Zvereva, Boris B. Dzantiev, Anatoly V. Zherdev

**Affiliations:** Research Centre of Biotechnology, A.N. Bach Institute of Biochemistry, Russian Academy of Sciences, Leninsky Prospect 33, 119071 Moscow, Russia; a.ivanov@fbras.ru (A.V.I.); dspopravko@mitht.ru (D.S.P.); safenkova@inbi.ras.ru (I.V.S.); zverevaea@yandex.ru (E.A.Z.); dzantiev@inbi.ras.ru (B.B.D.)

**Keywords:** meat adulteration, chicken additives, pig additives, cytochrome B, recombinase polymerase amplification, lateral flow assay, rapid test

## Abstract

Verifying the authenticity of food products is essential due to the recent increase in counterfeit meat-containing food products. The existing methods of detection have a number of disadvantages. Therefore, simple, cheap, and sensitive methods for detecting various types of meat are required. In this study, we propose a rapid full-cycle technique to control the chicken or pig adulteration of meat products, including 3 min of crude DNA extraction, 20 min of recombinase polymerase amplification (RPA) at 39 °C, and 10 min of lateral flow assay (LFA) detection. The cytochrome B gene was used in the developed RPA-based test for chicken and pig identification. The selected primers provided specific RPA without DNA nuclease and an additional oligonucleotide probe. As a result, RPA–LFA, based on designed fluorescein- and biotin-labeled primers, detected up to 0.2 pg total DNA per μL, which provided up to 0.001% *w*/*w* identification of the target meat component in the composite meat. The RPA–LFA of the chicken and pig meat identification was successfully applied to processed meat products and to meat after heating. The results were confirmed by real-time PCR. Ultimately, the developed analysis is specific and enables the detection of pork and chicken impurities with high accuracy in raw and processed meat mixtures. The proposed rapid full-cycle technique could be adopted for the authentication of other meat products.

## 1. Introduction

In modern society, falsifying the composition of food products by violating the declared recipe has become a serious problem [1,2,3,4]. The commercial interests of manufacturers lead to the use of cheaper substitutes for primary meat compounds. The consequences of these actions include the misinformation of consumers, the violation of religious and social norms, and health risks [5,6]. The manufacture of meat products is the branch of the food industry in which problems of falsification are most severe, and recent incidents related to the use of unauthorized sources of raw materials have caused notable public responses [7,8,9]. Among the various substituents used, two require systematic control. The confirmation of halal status, an essential element of which is control over the absence of deliberately or accidentally added pork, is in demand among a significant number of consumers for religious reasons [10,11,12,13]. To replace declared expensive types of raw meat materials with cheaper alternatives, poultry products are generally used, primarily chicken meat, which is inexpensive [14,15,16]. Therefore, much attention has been paid to tools for controlling the pork and chicken adulteration of meat products, in terms of both practical monitoring and the development of new techniques. The variety of methods used to solve these problems is extremely wide and includes the identification of specific biomarkers obtained by electrophoresis and chromatography [17,18,19] and assessment of the compositional characteristics of raw materials via microscopy and spectroscopy [20,21,22,23]. However, these methods are extremely laborious, characterized by low productivity, and require highly qualified personnel and expensive equipment that is available only to a limited number of centralized laboratories. Immunochemical methods of analysis, such as the enzyme-linked immunosorbent assay (ELISA) and lateral flow immunoassay (LFIA), are much less demanding in terms of instrumentation and skills, but the selectivity of antibodies is often insufficient to produce an unambiguous conclusion about the species of the used raw materials [24,25,26,27,28,29]. To date, the most actively used approach for controlling the raw material composition of food products is polymerase chain reaction (PCR) in its various iterations, including real-time PCR, multiplex PCR, etc. [30,31,32,33,34]. The high sensitivity and reliability of PCR results are advantages of this approach. However, the need for strictly fixed temperature cycling and the significant risk of errors due to the contamination of samples over the course of testing makes PCR suitable primarily for laboratory analysis. In other words, PCR is suitable for confirming conclusions, but simpler and more mobile methods of initial screening remain to be determined.

The aforementioned requirements are met by isothermal amplification methods. These methods retain PCR’s selectivity and sensitivity for nucleic acid detection but can be implemented with significant simplification of the operator’s actions and minimal instrumentation. To control the adulteration of meat products, approaches have been proposed based on different variants of isothermal amplification, and attention to these techniques has increased in recent years [27,35]. Loop-mediated isothermal amplification (LAMP) is the most popular method for rapid DNA detection [36,37,38,39,40,41]. These tests utilize fluorescence detection or color changing visualization to enable naked-eye detection. The lateral flow assay was also designed for the detection of meat-admixture LAMP products [42,43]. Recombinase polymerase amplification (RPA) is generally used to detect meat adulterants (chicken, duck, pig, etc.) with fluorescent and color detection [44,45] or a lateral flow test [46,47,48,49,50]. Other isothermal amplification methods have been employed for designing adulteration tests, including rolling circle amplification for horse-meat identification [51] and single primer-triggered isothermal amplification for chicken-meat detection [52]. However, existing models demonstrate the merits of certain novel approaches and do not offer a single procedure that combines the integration of accelerated sample preparation, isothermal amplification, and detection able to simplify all stages of real testing.

Considering the limitations of existing methods, the aim of this study was to propose and characterize a single protocol for the control of pork and chicken as priority adulterants. This integrated protocol combines (a) simple and rapid sample preparation; (b) a short amplification stage with a minimum number of reagents and manipulations; and (c) simple registration of the assay results, including its possible application without instrumentation. In this paper, the aforementioned three requirements were met by, respectively, by (a) the fast mechanical processing of raw materials without extraction and the quantitative isolation of DNA; (b) low-temperature amplification based on RPA and the design of special primers, accounting for the differences between this method and PCR; and (c) the use of lateral flow assay (LFA) with colloidal gold labels to visually detect the presence of target products in the reaction mixture.

## 2. Results and Discussion

### 2.1. Primer Design and Primary Verification by PCR

Several genes and other genome regions can be used for the taxonomic differentiation of biological samples. Some of these sites are highly repeated in genomes (short interspersed nuclear element (SINE) and long interspersed nuclear element (LINE) [53,54], whereas others are located in mitochondrial DNA, as copies of these sites can be found in high numbers in muscle tissues [55,56,57]. Genes and intergenic spacers of mitochondrial DNA are commonly used for species-specific assays because such spaces evolve rapidly within species evolution [58]. Authors of meat adulteration surveys have previously utilized the atp8 [59] and cytB [60,61] genes as targets (see the review in [62]). We selected the cytochrome b gene as a target for developing the RPA test based on the work in [61]. Sites of cytB that demonstrated high diversity upon multiple alignment were selected as potential targets for primer design (the sequences of cytB gene are presented in the Supplementary Materials, Section S1). The criteria for primer design were the absence of cross-dimerization among species, no self-dimer formation upon prediction, and a primer length of more than 25 nt for effective RPA. As a result, two forward primers (F3c and F4c) and two reverse primers (R3c and R3c) were selected for chicken, and four forward primers (F2p, F3p, F4p, and F5p) and three reverse primers (R3p, R4p, and R6p) were selected for pig. The selected primers are summarized in Table 1. Additionally, F1c/R1c and F1p/R1p pairs, recommended for the PCR detection of chicken and pig DNA in meat products, respectively [63], were chosen as references (Table 1). However, the possibility of stable self-dimers was predicted for the F1c primer. Thus, we modified (extended) the Fc1 primer to F2c, which made the primer more appropriate for RPA and reduced the probability of self-dimer formation. All forward primers were modified by biotin at the 5′ terminal, and reverse primers were modified by FAM at the 5′ terminal.

Real-time PCR was used as the first stage of evaluation for the designed RPA primers to recognize the obtained cytB gene. We verified five pairs for chicken cytB, including two combinations based on PCR primers (F1c–R1c, and F2c–R1c) and three pairs designed for the first time (F3c–R3c, F3c–R4c, and F4c–R4c) (see Supplementary Materials, Section S1). All pairs were able to detect purified cytB genes in the qPCR test. The obtained concentration dependencies of the cytB copies had a standard linear form and did not preclude any pairs from further experiments. The combination F2c–R1c demonstrated the greatest sensitivity in qPCR (Supplementary Materials, Section S2, Figure S1A). However, the manufacturer’s recommended RPA amplicon length (<800 bp) limited use of the F3c–R4c pair with a 1023 bp length (Table 2). Thus, four pairs (F1c–R1c, F2c–R1c, F3c–R3c, and F4c–R4c) were ultimately chosen for RPA–LFA.

For pig cytB, the F1p–R1p pair used earlier for PCR [63] and the nine pairs designed for the first time—F2p–R3p, F2p–R4p, F2p–R6p, F3p–R3p, F3p–R4p, F3p–R6p, F4p–R4p, F4p–R6p, and F5p–R6p—were proposed for qPCR. However, the manufacturer’s recommendation of RPA amplicon length (<800 bp) limited the use of F2p–R6p and F3p–R6p and reduced the tested list to eight pairs (Table 2). After refusing the primers that tend to form dimers, we selected four combinations (F3p–R3p, F3p–R4p, F4p–R6p, and F5p–R6p) and carried out a qPCR test (see Supplementary Materials, Section S2, Figure S1B). The F3p–R4p combination showed the least sensitivity in qPCR. Moreover, an amplicon with a size of 679 bp for F3p–R4p is worse when detected by lateral flow test strips. Therefore, for further investigations in RPA–LFA, only four pairs (F1p–R1p, F3p–R3p, F4p–R6p, and F5p–R6p) were retained.

### 2.2. Verification of Designed Primers for the Specificity of RPA–LFA

**Figure 1:**
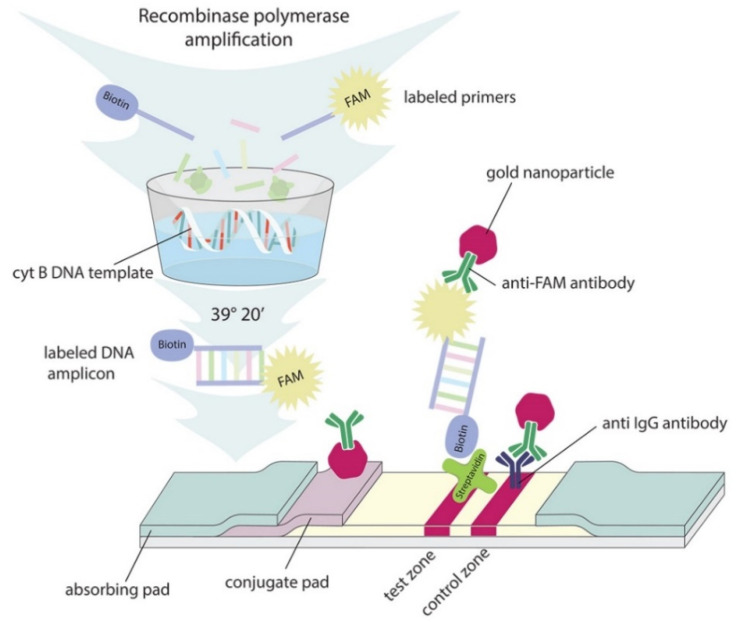
The scheme of RPA–LFA used for detection of either chicken or pig DNA. RPA was performed in the presence of labeled primers using cytochrome b DNA as a template. RPA generated labeled amplicons that were applied to the lateral flow strips. In the presence of the target DNA, an immune complex of a FAM-labeled terminal with an anti-FAM–GNP conjugate was formed as the labeled amplicons passed along the strip. The following streptavidin–biotin reaction occurred in the test zone and caused its colorization via GNP inclusion.

The primer pairs approved by qPCR were used in RPA with subsequent detection on a lateral flow test strip. As a result, RPA double-stranded DNA amplicons labeled with FAM on one end and biotin on the other end were formed. To recognize, bind, and detect the labeled amplicons, we obtained lateral flow test strips using a type of “sandwich” assay. Streptavidin immobilized in the test zone bound biotin at one end of the amplicon, and an anti-FAM–gold nanoparticle (GNP) conjugate bound FAM at the opposite end of the amplicon. In this way, a triple complex was formed in the test zone, including the streptavidin–amplicon-labeled biotin and the FAM–anti-FAM–GNP conjugate. The presence of amplicons in the sample resulted in two colored bands (in the test and control zones) due to the GNPs (the test-strip scheme is shown in (Figure 1); in the absence of amplicons in the sample, only one colored band was formed in the control zone.

**Figure 2:**
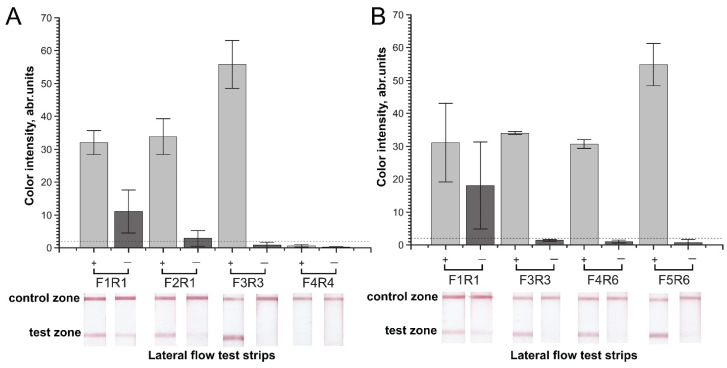
Effect of the primer pair in RPA–LFA for total DNA isolated from chicken (**A**) or pig (**B**) meat and nontarget DNA. Color intensities of the test zones and corresponding scans of the test strips. “+”—positive meat control, “−” negative—control (nontarget DNA). The dashed line represents the signal visible to the naked eye (2 a.u.).

We observed a false-positive signal in the RPA–LFA of the selected pairs due to cross-dimer formation. Comparison of the RPA–LFA results for total DNA isolated from chicken or pig meat and nontarget DNA demonstrated significant differences in the signal-to-noise values for the different primer pairs (Figure 2). For chicken, the F1c–R1c pair demonstrated a pronounced false-positive signal (Figure 2A). The pair that was most sensitive in PCR (Figure S1A), F2c–R1c, gave a low signal. The mean signal of the F2c–R1c combination (2.9 a.u.) was higher than the limits of naked-eye detection (2 a.u.), and the high levels of dispersion in the samples with nontarget DNA led to the exclusion of these primers from further experiments. Two other combinations of primers demonstrated non-significant false-positive signals that were below a visible level (2 a.u.). However, the F4c–R4c pair did not demonstrate a positive signal in RPA–LFA with the target total chicken DNA (Figure 2A). Only the F3c–R3c pair was able to form a highly pronounced signal in the RPA of the total DNA from chicken meat and, besides, it did not form cross-dimers and produce a false-positive signal. Considering these features, we chose the F3c–R3c pair for further applications.

For pig, four selected pairs (F1p–R1p, F3p–R3p, F4p–R6p, and F5p–R6p) also showed differences in RPA–LFA (Figure 2B). The F1–R1 pair demonstrated a tangible false-positive signal, as expected. The high dispersion of F1p–R1p in both RPA tests precluded use of the pair in subsequent tests. Other pairs caused false-positive signals with significantly lower levels of visibility. We ultimately chose F5p–R6p, which provided the most pronounced signal-to-noise value. However, F3p–R3p and F4p–R6p also presented good signal-to-noise values and were used as reserve variants.

Previously designed primers for PCR (F1c–R1c and F1p–R1p) appeared to be inappropriate in RPA–LFA. Despite providing good sensitivity in the performed PCR tests with the cytB target DNA (Figure S1), these primers demonstrated pronounced false-positive signals in RPA–LFA (Figure 2). F1p–R1p was predicted to form a stable cross-dimer, carrying FAM and biotin, which is recognized as a target in LFA. The formation of this dimer is less possible in PCR because a high temperature is recommended for primer annealing. In the case of F1c–R1c, the dimers were not predicted. The observed difference demonstrated the need for experimental RPA verification of the designed primers for RPA, despite successful primary verification by PCR.

**Figure 3:**
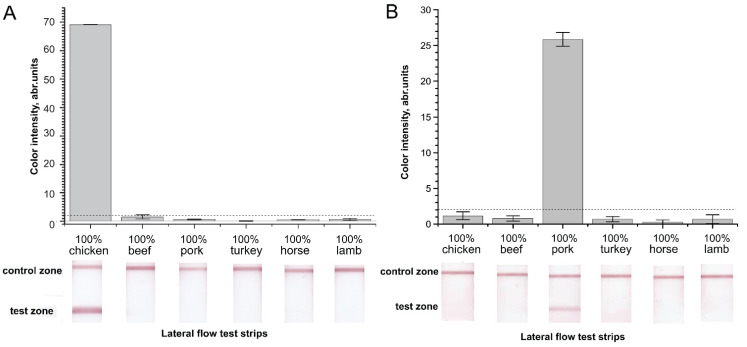
Specificity of RPA–LFA with the selected primers for total DNA extracted from meats of different origin. Color intensities of the test zone and corresponding scans of the test strips after developing the RPA–LFA. (**A**). RPA–LFA for chicken meat detection, (**B**). RPA–LFA for pork detection. The dashed line represents the signal visible to the naked eye (2 a.u.).

The next selection stage involved confirming the absence of cross-reactivity in the RPA–LFA of the previously selected primer pairs. We checked whether the selected aforementioned primer pairs produced a nonspecific signal in the RPA–LFA of nontarget meat DNA (Figure 3). The chicken F3c–R3c and pig F5p–R6p pairs were tested with 1 µg of total DNA from six species (chicken, beef, pork, turkey, horse, and lamb) to estimate cross-reactivity. The chicken F3c–R3c pair demonstrated a highly specific signal in RPA–LFA (Figure 3A). The RPA–LFA for the nontarget DNA of pork, turkey, horse, and lamb produced signals in the test zones significantly below the level of visibility by the naked eye (<2 a. u). For the beef total DNA, the mean signal was below the cut-off limit (1.6 a.u.), but its high dispersion was not able to discriminate the signal as negative. On the other hand, the positive signal of the chicken total DNA after amplification was 43-fold higher than the signal of beef DNA after amplification.

The pig-specific primer pair F5p–R6p also demonstrated a specific signal. All non-pork total DNA demonstrated signals significantly lower than 2 a.u. (Figure 3B). The positive signals surpassed the signals of the other samples by 25–100-fold. The obtained results confirmed the optimal selection of the primers for the specific RPA–LFA of chicken and pig additions in meat products. Thus, the designed and selected primers for the detection of chicken and pig cytochrome b demonstrated neither false-positive nor false-negative activity and could be applied to the lateral flow detection of RPA products.

### 2.3. Sensitivity of RPA–LFA

**Figure 4:**
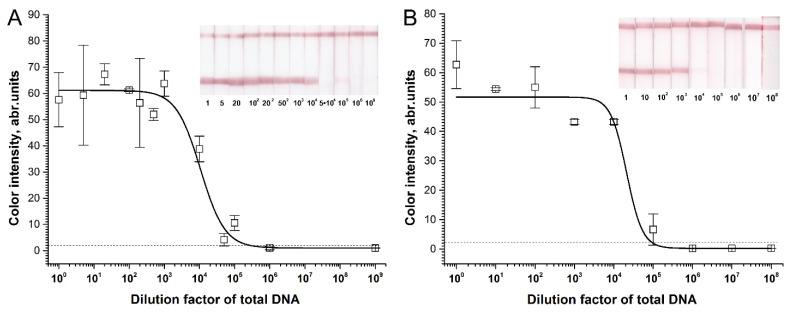
Dependences of color intensity in the test zone after RPA–LFA for the dilution of total DNA from chicken (**A**) and pork (**B**) meat. The scans of the strips correspond to the plotted values. The dashed line represents the signal visible to the naked eye (2 a.u.).

To estimate the analytical characteristics of the test system that was developed, we performed RPA–LFA on the diluted purified total genomic DNA of the chicken and pig. Previously selected chicken F3c–R3c and pig F5p–R6p primers were applied in this RPA–LFA. Quantitative PCR of the same amount of total DNA was used as the reference method. The sensitivity of the assay was determined by serial 10-fold dilutions (starting from 1 µg target total DNA per reaction) of total extracted DNA in salmon sperm DNA. The RPA–LFA of the total DNA demonstrated a pronounced signal in the test zone that appeared at up to 10^4^ dilution and provided a short linear response of 10^3^–10^5^ for the dilutions (Figure 4). However, both the chicken and pork total DNA were visually detectable at up to a 100,000-fold dilution of the initial DNA (Figure 4), which was equal to 10 pg of total DNA in the RPA reaction or 0.2 pg/µL of the total DNA in the reaction mix. Real-time qPCR was also performed on the serially diluted total DNA of chicken and pig with different primers including F3c–R3c for chicken and F5p–R6p for pig (Supplementary Materials, Section S2, Figure S2). The detection limits of qPCR were 1 pg target DNA per reaction or 0.1 pg/µL for chicken (Figure S2A) and pig (Figure S2B). Thus, the developed RPA–LFA and qPCR had highly similar detection limits. The qPCR test exceeded the developed RPA–LFA by only twofold. We fit the experimental concentration dependence in semi-logarithmic axes using a sigmoidal curve. According to the approximation curves, the detection limit of F3c–R3c was 1.9 × 10^5^ dilution, with 9.1 × 10^4^ dilution as the limit for F5p–R6p. These values were close to the limits of naked-eye detection.

We estimated the approximate number of the cytB gene copies in total DNA, comparing the Ct of the calibration curve of purified cytB genes (Figure S1A,B) with the qPCR for total DNA (Figure S2A,B). In this way, 1 pg of the extracted chicken DNA contained about 100 copies of cytB, and 1 pg of the extracted pig DNA contained 10 copies of cytB. Thus, the sensitivity of RPA–LFA was twenty copies of chicken cytB per µL and two copies of pig cytB per µL.

These results were used to estimate the limit of detection for meat adulteration. The developed test can detect 10^5^ diluted total DNA or 0.001% of the initial total DNA. Thus, we set the estimated limit of adulteration sensitivity at 0.001%. Despite the possibility that the presence of nontarget DNA in real adulterated preparations could diminish the sensitivity, the value was convenient as a point of comparison with the theoretical sensitivity of other tests. The RPA–LFA (with TwistDx nfo kit) developed by Lin et al. [47] was based on the NDL4 (chicken) and ND1 (pig) genes of mtDNA (not cytB) and provided 20 pg of total DNA per µL as the detection limit, which resulted in 0.1% *w*/*w* of meat-component identification. Moreover, the second known RPA–LFA with the TwistDx nfo kit amplified the microsatellite site of the Mangalica pig and was able to detect 170 pg of total DNA per µL [48], which resulted in 0.85% *w*/*w*. These data demonstrated the higher sensitivity potential of our developed test.

### 2.4. Verification of the RPA–LFA with Meat Samples

**Figure 5:**
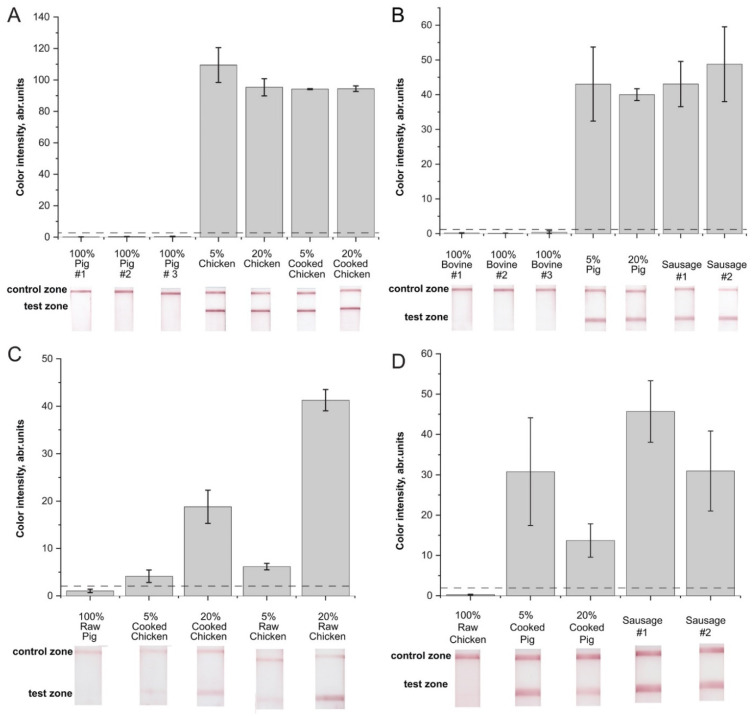
RPA–LFA of meat samples. (**A**). Detection of chicken meat in samples after accurate DNA extraction. (**B**). Detection of pig meat in samples after accurate DNA extraction. (**C**). Detection of chicken meat in samples after rapid DNA extraction. (**D**). Detection of pig meat in samples after rapid DNA extraction. Number of repeats: 2. The dashed line represents the signal visible to the naked eye (2 a.u.).

The applicability of the developed RPA–LFA in detecting pork and chicken adulteration was evaluated based on the analysis of mixed meat samples and samples of processed meat containing components of target and nontarget animals. We took samples containing 5% and 20% chicken (or pig) meat additives. Moreover, we tested the preheated (72 °C) composition of meat in 5% and 20% chicken additives and tested 25% pig fatback in sausages as processed meat products. First, we used the conventional longform method of DNA extraction (see Section 3.3) to isolate accurately extracted DNA. As a result, strong positive results and corresponding high values of color intensities were obtained for the test zones (Figure 5A,B). Although heat treatment impairs the extraction of DNA from meat samples according to [64], our results showed no differences based on sample composition at high concentrations of the target meat (*w*/*w*) (see Figure 5A,B). The negative control (nontarget meat) showed no visible signal in the test zone for both RPA–LFA tests. Testing the same samples with qPCR (data are presented in the Supplementary Materials, Section S3) confirmed the positive and negative results obtained with the RPA–LFA.

For a simple and rapid means of sample preparation, we used the second approach (see Section 3.3) to provide crude DNA extracts after rapid homogenization. The obtained RPA–LFA results are presented in Figure 5C,D. After rapid homogenization, the RPA–LFA test also specifically detected chicken or pig meat in the corresponding samples. The control nontarget samples demonstrated signals significantly below the limits of naked-eye detection for both RPA–LFAs. Moreover, samples containing chicken meat demonstrated positive signals in RPA–LFA significantly above visible levels (Figure 5C). The RPA–LFA signals of chicken samples upon use of the crude extract were 2.5–20-fold lower than the signals after analyzing accurately extracted DNA (Figure 5A,C). Differences between the chicken sample with heat processing and that without processing were visible only for samples containing 20% chicken (see Figure 5C). Dispersion of the detection results of the crude extracts appeared higher than dispersions after accurate DNA extraction. Samples containing pork meat also presented positive signals after crude extraction (Figure 5D). The decline in the signals was less than 2.5-fold when comparing accurately extracted DNA from the same samples (Figure 5B,D). However, high variance in the signals between repeats was observed after rapid DNA extraction.

Therefore, crude extraction for 3 min reduced the sensitivity of the analysis due to incomplete DNA extraction but significantly saved analysis time. Crude extraction provided a five-fold reduction in overall analysis time. As a result of the high sensitivity of the developed RPA–LFA, the crudely extracted meat DNA was used for the adulteration test. The approach of crude extraction has only been used once for meat samples in [48] for the RPA–LFA identification of Mangalica pig. However, the identification of Mangalica pig was significantly less sensitive (0.85% *w*/*w*) than the developed RPA–LFA (0.001% *w*/*w*), which limited the application of crude extraction.

The experiments presented in this section compare two strategies for the adulteration assay. The first strategy involves the thorough extraction of DNA; this method requires considerable time but provides higher signals and could be applied when risk of low content of contaminants is considered as a key parameter of an assay. The detection of minor adulterations is also important due to ethical and religious concerns. The second strategy relies on the rapid detection of adulteration. In this way, the sensitivity of the assay is considered to be a less important parameter. This type of test could be performed when relatively high thresholds for permissible contamination are set by regulations—for example, for the verification of certified meat products. The developed RPA–LFA can be applied under both strategies, depending on the aim of the assay.

### 2.5. Comparison of the Developed Technique to Control Chicken or Pig Adulteration and Other RPA-Based Assays

The developed technique to control chicken or pig adulteration included accelerated sample preparation (3 min at room temperature), RPA (20 min at 39 °C), and lateral flow test-strip detection (10 min at room temperature). We compared other known RPA-based assays against the technique developed in the present study (Table 3). Ultimately, our RPA-based test system achieved sensitivity of 0.2 pg/μL for the total DNA of chicken or pig, which falls within the range of sensitivity of other RPA-based tests (0.17–20 pg/μL). At the same time, the extraction methods for most of the methods presented in Table 2 are based on long-term DNA extraction using commercial kits, requiring an amplification time of 20 to 205 min. Our technique is highly compatible with rapid extraction over 3 min. Due to this technique’s high sensitivity, crudely extracted DNA can be used. In this work, we used the cytB gene for the first time to develop an RPA-based test. Notably, the selected DNA targets and optimized primers removed the need for instrumental detection using, e.g., PCR or real-time RPA [44], DNA nuclease and an additional oligonucleotide probe comprising tetrahydrofuran for RPA [47,48], or the high-temperature hybridization of labeled probes [46]. Therefore, the proposed method offers the best combination of sensitivity, simplicity, and speed for RPA-based assays to control chicken and/or pig adulteration.

## 3. Materials and Methods

### 3.1. Reagents

Kits for RPA were manufactured by TwisDx (Maidenhead, UK). Biotin- and fluorescein (FAM)- labeled oligonucleotides were synthesized by Syntol (Moscow, Russia). Proteinase K, a mix for PCR containing SYBR Green I, dNTP, Taq polymerase, and a DNA purification kit were purchased from Evrogen (Moscow, Russia). The nitrocellulose membrane CNPC12, PT R5 fiberglass membrane, sample pad membrane GFB-R4, and absorbent pad AP045 were produced by Advanced Microdevices (Ambala Cantt, India). Mouse monoclonal IgG specific to fluorescein (anti-FAM) was produced by Bialexa (Russia). Recombinant streptavidin and goat anti-mouse IgG were produced by Imtek (Russia). HAuCl_4_, bovine serum albumin (BSA), and lyophilized salmon-sperm DNA were purchased from Sigma-Aldrich (St. Louis, MO, USA). Salts, buffers, organic solvents, and other low-molecular-weight organic compounds were of analytical grade and purchased from different commercial retailers.

### 3.2. Meat Samples

Meat samples (chicken (*Gallus gallus*), bovine (*Bos taurus*), pig (*Sus scrofa*), turkey (*Meleagris gallopavo*), and mutton (*Ovis aries*)) were obtained from local stores. Horse meat (*Equus caballus*), meat mixes, and sausages were obtained from the V. M. Gorbatov Federal Research Center for Food Systems of Russian Academy of Sciences (Moscow, Russia). We tested the following meat mixes:

- Mix N1: 5% pig; 4.4% chicken; 90.6% bovine;
- Mix N2: 20% pig; 17.8% chicken; 62.2% bovine;
- Mix N3: 5% chicken muscle; 5% chicken skin; 90% pig;
- Mix N4: 20% chicken; 17.8% chicken skin; 62.2% pig.
- Additionally, mixes N3 and N4 were temperature-treated up to 72 °C. The sausages were of two types:
- Sausage N1, comprising 40% pig muscles, 25% pig fatback, and 35% bovine according to [65];
- Sausage N2, comprising 25% pig fatback and 75% bovine according to [66].

**Table 3:** Comparison of RPA-based tests for chicken and pig adulteration.

Meat	Target Sequence	Sensitivity of DNA	Adulteration, %	Detection Method	Time of Amplification/Time of LFA	LoD of PCR	Extraction Method	Extraction Time, Min	Ref.
Mangalica pig	Microsatellite locus	0.17 ng/µL = 50 copies/reaction (1 copy/µL)	ND	LFA of RPA-nfo product	30/5	NA	Wizard^®^ kit (Promega, Madison, WI, USA) ǁ DNAreleasy^®^ (Nippon Genetics Europe, Düren, Germany) ǁ crude homogenization in water	>180 ǁ 15 ǁ <5	[48]
Chicken	D-loop	10^4^ copies/µL = 20 pg total DNA/reaction	1	SYBR Green I coloration	30	100 copies/µL	Universal Genomic kit (CWBIO, Taizhou, China)	85–205	[45]
Pig		10^3^ copies/µL = 20 pg total DNA/reaction	1			100 copies/µL			
Pig	ND2	1.23 pg total DNA/reaction = 10 copies/reaction	0.1	Real-time fluorescence by mobile equipment	15	NA	QIAamp^®^ DNA Mini Kit (Qiagen, Hilden, Germany)	20	[44]
Pig	D-loop	10 pg total pig DNA	1	Probe hybridization (with stage at 95 °C for 5 min) followed by LFA	40/8		DNeasy blood and tissue kit (Qiagen, Hilden, Germany)	20–60	[46]
Chicken	NDL4	10 copies (plasmid)/µL, 20 pg total DNA/µL	1	LFA of RPA-nfo product	20/4	Pos/neg test	gDNA extraction kit (Tiangen, Beijing, China)	60	[47]
Pig	ND1	10 copies (plasmid)/µL, 20 pg total DNA/µL							
Chicken	Cyt B	0.2 pg total DNA/µL = 20 copies/ µL	5	LFA of TwisDx basic products	20/10	0.1 pg total DNA/µL	Salt method ǁ Crude homogenization	120 ǁ <3	This study
Pig		0.2 pg total DNA/µL = 2 copies/µL				0.1 pg total DNA/µL			

### 3.3. Meat Processing and DNA Extraction

We used two methods of DNA extraction from the meat samples: the salt method according to Yalcinkaya et al. [67], with some modifications, and rapid extraction with minimal processing. The salt method involved homogenization of a 50 mg sample via grinding in a mortar and dissolving the sample in 400 µL of lysis buffer (10 mM Tris-HCl, pH 8.0, with 2 mM EDTA, 0.4 M NaCl) and 40 µL of 20% (*w*/*v*) SDS. The tubes were vortexed intensively. Then, 5 µL of 20 mg/mL proteinase K was added, and the mixture was incubated for 1 h at 65 °C. Then, we added 300 µL of 6 M NaCl and vortexed the mixture for 30 s. After centrifugation for 30 min at 10,000 *g*, the supernatant was transferred into a clean microcentrifuge tube. Next, an equal volume of isopropanol was added to the supernatant. The tube mixture was further mixed by shaking the tube up and down and was then incubated at −20 °C for 10 min. After centrifugation for 20 min at 16,000 *g*, the supernatant was removed, and the pellet was dried. The pellet was dissolved in 100 µL of TE buffer (10 mM Tris-HCl, pH 8.0, with 2 mM EDTA) or mQ water. The concentration of the obtained DNA was measured on a NanoDrop ND-2000 analyzer (USA).

Rapid DNA extraction from the meat samples was performed via crude homogenization in a mortar with 100 mg of the samples in a 1 mL buffer containing 10 mM Tris HCl, pH 8.0, with 50 mM NaCl for 1 min, followed by separation of the liquid phase with centrifugation at 4000 *g*.

### 3.4. Primer Design

We performed multiple alignment of the cytochrome b gene from six species with Gene Bank accession numbers of X56295.1 (*S. scrofa*), KT151960.1 (*B. taurus*), DQ223538.1 (*E. caballus*), X56284.1 (*O. aries*), L08376.1 (*G. gallus*), and L08381.1 (*M. gallopavo*) using the online software MUSCLE (www.ebi.ac.uk/Tools/msa/muscle/, accessed on 10 November 2021) and BLAST (https://blast.ncbi.nlm.nih.gov/Blast.cgi, accessed on 10 November 2021). Primers for the RPA of pig and chicken samples were designed using OligoCalc (www.biotools.nubic.northwestern.edu/OligoCalc.html, accessed on 10 November 2021) and the Multiple Primer Analyzer ThermoFisher online software. Only primer pairs that had no predicted dimers were selected.

### 3.5. Synthesis of Cytochrome b Genes

Genes of cytochrome b (cytB) from *G. galus* and *S. scrofa* were amplified from the total DNA extracted from the corresponding meat samples. The following primers were used: cytB_chicken_forward ATGGCACCCAACATTCGA, cytB_chicken_reverse TTAGTAGTTGAGTATTTTG, cytB_pig_forward ATGACCAACATCCGAAAATCAC, and cytB_pig_reverse TCTTCATTTTAATAGGTTGTT. The PCR mix contained 200 nM primers, 100 nM of each dNTP, 5 units of Taq polymerase in an appropriate buffer, and 1 µg of total DNA. The PCR cycle of chicken cytB involved a denaturation stage at 95 °C for 30 s, a primer annealing stage at 55 °C for 30 s, and an elongation stage at 72 °C for 60 s. The PCR cycle of pig cytB involved a denaturation stage at 95 °C for 30 s, a primer annealing stage at 50 °C for 30 s, and an elongation stage at 72 °C for 60 s. For both genes, 40 PCR cycles were performed. The amplified cytB genes were purified by electrophoresis in 1% agarose gel and extracted with a DNA purification kit. The concentration of the obtained DNA was measured on the NanoDrop ND-2000 (Thermo Fisher Scientific, Waltham, MA, USA).

### 3.6. Quantitative Real-Time PCR

Different concentrations of the target DNA were added to the solution containing 200 nM of the designed primer pairs (Table 1) and a commercial SYBR Green I–qPCR mix (Evrogen). The final volume of the reaction was 10 µL. Real-time quantitative PCR was performed using a LightCycler 96 device (Roche, Switzerland). The analysis required 45 cycles, with the detection of fluorescence performed after each cycle. The PCR cycle involved a denaturation stage at 95 °C for 30 s, a primer annealing stage at 66 °C for 30 sec, and an elongation stage at 72 °C for 30 s. The threshold cycle (Ct) was computed automatically using the LightCycler software (Roche, Basel, Switzerland).

### 3.7. Preparation of Lateral Flow Test Strips

Gold nanoparticles (GNPs) were synthesized according to the modified Frens method by reducing HAuCl_4_ with sodium citrate (see Supplementary Materials, Section S4) [68]. The GNP solution with 1.0 absorbance units at 520 nm (OD520) was adjusted to pH 9.0. Then, anti-FAM antibodies were added to the GNP solution at a final concentration of 13 μg/mL and stirred for 1 h at 20 °C. The surface of the conjugates was blocked by adding 0.25% BSA (*m*/*w*) followed by stirring for 30 min. Unbound proteins were separated via precipitation of the GNP–antibody conjugate using centrifugation at 14,000 *g* for 30 min.

The lateral flow test strips used to detect RPA amplicons (double-stranded DNA labeled with FAM-biotin) were prepared according to Safenkova et al. [69]. The multimembrane composites were assembled using plastic supports with a CNPC-12 nitrocellulose membrane, PT R5 glass fiber membrane for the conjugate (conjugate pad), GFB R4 sample pad, and AP045 absorbent pad. Streptavidin was dispensed on the test zone, and goat anti-mouse antibodies were dispensed on the control zone using an IsoFlow Dispenser (Imagene Technology, St. Lebanon, NH, USA) with a dispersion rate of 0.15 μL per mm membrane; both used 1 mg/mL in 50 mM potassium phosphate buffer, pH 7.4, with 100 mM NaCl (PBS), glycerol 5%, and 0.03 % NaN_3_. The conjugate of GNP–anti-FAM antibodies (OD 520 = 4.0) was dispensed at 3.2 μL per 1 mm strip width on a PT R5 membrane. The multimembrane composites were dried after dispensing at 20 °C overnight and then cut on 3.5 mm strips using an Index Cutter-1 automatic guillotine (Arista Biologicals, Allentown, PA, USA) and packed in laminated aluminum foil bags with silica gel using an FR-900 continuous band sealer (Dingli Packing Machinery, Wenzhou, China).

### 3.8. RPA–LFA Test

An RPA TwistDx basic kit was used for the RPA according to the manufacturer’s protocol with modifications. First, 300 nM FAM- and biotin-labeled primers were added to the rehydration buffer. Then, 5 μL of the extracted total DNA was added. For negative controls, nontarget DNA (salmon sperm DNA) or total DNA was extracted from nonspecific meat products. For crude meat extraction, 10 μL of DNA extract was added. During the final stage, 14 mM magnesium acetate was added to start the reaction, which was carried out at 39 °C for 20 min in a T100 Thermal Cycler (BioRad, Hercules, CA, USA). For RPA–LFA, after the end of the reaction, 5 μL of the RPA mix solution was added to 55 μL PBS and used as a sample for LFA. Then, the test strip was submerged into the sample, and qualitative results were visually determined after 10 min. The quantitative values were obtained from color density after scanning the test strips using a CanoScan LiDE 90 scanner (Canon, Tokyo, Japan) and processed with the TotalLab TL120 software (Nonlinear Dynamics, Newcastle upon Tyne, UK). Statistical analyses (mean and standard deviation) and curve fitting with the four-parameter logistic function were performed using the OriginPro 9.1 software (OriginLab, Northampton, MA, USA).

## 4. Conclusions

Methodologies to identify additives in meat are developing beyond conventional PCR-based approaches. The use of an isothermal amplification approach, such as RPA, and a fast detection tool, such as LFA, is increasingly important considering the latest developments. In this regard, it is critical to find comprehensive solutions to control the adulteration of meat products, integrating sample preparation, amplification, and rapid detection. Our results show that DNA targets and primers optimized for PCR should not be used for RPA amplification. In addition, the choice of gene is of great importance, as our application of CytB allowed us to achieve high sensitivity (0.001% *w*/*w* or 0.2 pg total DNA/μL). Moreover, this choice facilitated the use of a basic RPA kit without DNA nuclease and an additional oligonucleotide probe containing tetrahydrofuran. In this work, only the primers fluorescein and biotin were used. The application of crude extraction in the RPA–LFA protocol reduced the sensitivity of the analysis and decreased the sample preparation time to 3 min. Therefore, we not only developed an RPA–LFA test but also proposed a rapid full-cycle 33 min technique to control meat adulteration. This technique could be applied to processed meat products or meat after heating. The proposed solutions will be useful for developing new rapid tools for identifying meat components even in trace quantities.

## Figures and Tables

**Table 1:** Sequences of primers used.

Species	Name	Sequences (5′ to 3′)	Length	Modification of 5′
*Galus galus*	F1c	TCACATCGGACGAGGCCTA	19	Biotin
	R1c	GGAATGGGGTGAGTATGAGAGTT	23	FAM
	F2c	TCACATCGGACGAGGCCTATACTAC	25	Biotin
	F3c	CCTATTAGCAGTCTGCCTCATGACC	25	Biotin
	R3c	GAGGCGCCGTTTGCGTGGAGATTCC	25	FAM
	F4c	CTTCAAAGACATTCTGGGCTTAACTC	26	Biotin
	R4c	ATTTTGTTTTCTAGTGTTCCGATTGT	26	FAM
*Sus scrofa*	F1p	GACCTCCCAGCTCCATCAAACATCTCATCATGATGAAA	38	Biotin
	R1p	GCTGATAGTAGATTTGTGATGACCGTA	27	FAM
	F2p	AACAACAGCTTTCTCATCAGTTACA	25	Biotin
	F3p	AAATTACGGATGAGTTATTCGCTATC	26	Biotin
	R3p	GTGCAGGAATATGAGATGTACGGCT	25	FAM
	F4p	AAAGACATTCTAGGAGCCTTATTTA	25	Biotin
	R4p	TAGGATGGAGGCTACTAGGGCCAAC	25	FAM
	F5p	AGCCTCCATCCTAATCCTAATTTTA	25	Biotin
	R6p	ATAGGTTGTTTTCGATGATGCTAGTG	26	FAM

**Table 2:** Length (bp) of possible amplification product with the selected primers.

Chicken (*G. galus*)					
	F1c	F2c	F3c	F4c	
R1c	431	431	614	44	
R3c	NA	NA	159	NA	
R4c	840	840	1023	453	
**Pig (*S. scrofa*)**					
	**F1p**	**F2p**	**F3p**	**F4p**	**F5p**
R1p	348	279	273	NA	NA
R3p	546	427	385	NA	NA
R4p	840	721	679	219	NA
R6p	107	953	911	415	246

NA: non-available.

## Data Availability

The data presented in this study are available on request from the corresponding author.

## Supplementary Materials

The following are available online: The Supporting Information file (PDF) contains the following sections: Section S1. Sequences of DNA amplicons and primers for cytochrome B of chicken and pig; Section S2. Characterization of primer pairs with real-time PCR; Section S3. Verification of the developed test with meat samples; Section S4. Synthesis of gold nanoparticles.
