# Supplementary material for: Rapid Full-Cycle Technique to Control Adulteration of Meat Products: Integration of Accelerated Sample Preparation, Recombinase Polymerase Amplification, and Test-Strip Detection
Source: Molecules. 2021 Nov 11;26(22):6804. doi: 10.3390/molecules26226804 (PMC8622786; doi:10.3390/molecules26226804)
Supplement: Supplementary file 1 [file molecules-26-06804-s001.zip › molecules-1432948-supplementary.pdf]

Supplementary Materials

Rapid full-cycle technique to control adulteration of meat products: Integration of accelerated sample preparation, recombinase polymerase amplification, and test-strip detection

Alexandr V. Ivanov, Demid S. Popravko, Irina V. Safenkova, Elena A. Zvereva, Boris B. Dzantiev, Anatoly V. Zherdev*

A.N. Bach Institute of Biochemistry, Research Centre of Biotechnology of the Russian Academy of Sciences, Leninsky prospect 33, 119071 Moscow, Russia

*Correspondence: zherdev@inbi.ras.ru; Tel.: +7-495-954-2804

CONTENT

Section S1. Sequences of DNA amplicons and primers for cytochrome B of chicken and pig 2

Section S2. Characterization of primer pairs with real-time PCR 4

Section S3. Verification of the developed test with meat samples 6

Section S4. Synthesis of gold nanoparticles 7

Section S1. Sequences of DNA amplicons and primers for cytochrome B of chicken and pig

Cyt B chicken:

ATGGCACCCAACATTCGAAAATCCCACCCCCTACTAAAAATAATTAACAACTCCCTA
ATCGACCTCCCAGCCCCATCCAACATCTCTGCTTGATGAAATTTCGGCTCCCTATTAG
CAGTCTGCCTCATGACCCAAATCCTCACCGGCCTACTACTAGCCATGCACTACACAG
CAGACACATCCCTAGCCTTCTCCTCCGTAGCCCACACTTGCCGGAACGTACAATACG
GCTGACTCATCCGGAATCTCCACGCAAACGGCGCCTCATTCTTCTTCATCTGTATCTT
CCTTCACATCGGACGAGGCCTATACTACGGCTCCTACCTCTACAAGGAAACCTGAAA
CACAGGAGTAATCCTCCTCCTCACACTCATAGCCACCGCCTTTGTGGGCTATGTTCTC
CCATGGGGCCAAATATCATTCTGAGGGGCCACCGTTATCACAAACCTATTCTCAGCA
ATTCCCTACATTGGACACACCCTAGTAGAGTGAGCCTGAGGGGGATTTTCAGTCGAC
AACCCAACCCTTACCCGATTCTTCGCTTTACACTTCCTCCTCCCCTTTGCAATCGCAG
GTATTACTATCATCCACCTCACCTTCCTACACGAATCAGGCTCAAACAACCCCCTAG
GCATCTCATCCGACTCTGACAAAATTCCATTTCACCCATACTACTCCTTCAAAGACA
TTCTGGGCTTAACTCTCATACTCACCCCATTCCTAACACTAGCCCTATTCTCCCCCAA
CCTCCTAGGAGACCCAGAAAACTTCACCCCAGCAAACCCACTAGTAACCCCCCCAC
ATATCAAACCAGAATGATATTTTCTATTCGCCTATGCCATCCTACGCTCCATCCCCAA
CAAACTTGGAGGTGTACTAGCCCTAGCAGCCTCAGTCCTCATCCTCTTCCTAATCCC
CTTCCTCCACAAATCTAAACAACGAACAATAACCTTCCGACCACTCTCCCAAACCCT
ATTCTGACTTCTAGTAGCCAACCTTCTTATCCTAACCTGAATCGGAAGCCAACCAGT
AGAACACCCCTTCATCATCATTGGCCAAATAGCATCCCTCTCTTACTTCACCATCCTA
CTTATCCTCTTCCCCACAATCGGAACACTAGAAAACAAAATACTCAACTACTAA

Primer annealing sites marked by: underline for F1c, yellow for F2c, green for F3c, cyan for F4c, double underline for R1c, magenta for R3c, red for R4c

Cyt B pig:

ATGACCAACATCCGAAAATCACACCCACTAATAAAAATTATCAACAACGCATTCATT
GACCTCCCAGCCCCCTCAAACATCTCATCATGATGAAACTTCGGTTCCCTCTTAGGC
ATCTGCCTAATCTTGCAAATCCTAACAGGCCTGTTCTTAGCAATACATTACACATCA
GACACAACAACAGCTTTCTCATCAGTTACACACATTTGTCGAGACGTAAATTACGGA
TGAGTTATTCGCTATCTACATGCAAACGGAGCATCCATATTCTTTATTTGCCTATTCA
TCCACGTAGGCCGAGGTCTATACTACGGATCCTATATATTCCTAGAAACATGAAACA
TTGGAGTAGTCCTACTATTTACCGTTATAGCAACAGCCTTCATAGGCTACGTCCTGC
CCTGAGGACAAATATCATTCTGAGGAGCTACGGTCATCACAAATCTACTATCAGCTA
TCCCTTATATCGGAACAGACCTCGTAGAATGAATCTGAGGGGGCTTTTCCGTCGACA
AAGCAACCCTCACACGATTCTTCGCCTTCCACTTTATCCTGCCATTCATCATTACCGC
CCTCGCAGCCGTACATCTCCTATTCCTGCACGAAACCGGATCCAACAACCCTACCGG
AATCTCATCAGACATAGACAAAATTCCATTTCACCCATACTACACTATTAAAGACAT
TCTAGGAGCCTTATTTATAATACTAATCCTACTAATCCTTGTACTATTCTCACCAGAC
CTACTAGGAGACCCAGACAACTACACCCCAGCAAACCCACTAAACACCCCACCCCA
TATTAAACCAGAATGATATTTCTTATTCGCCTACGCTATTCTACGTTCAATTCCTAAT
AAACTAGGTGGAGTGTTGGCCCTAGTAGCCTCCATCCTAATCCTAATTTTAATGCCC
ATACTACACACATCCAAACAACGAGGCATAATATTTCGACCACTAAGTCAATGCCTA
TTCTGAATACTAGTAGCAGACCTCATTACACTAACATGAATTGGAGGACAACCCGTA

GAACACCCGTTCATCATCATCGGCCAACTAGCCTCCATCTTATACTTCCTAATCATTC
TAGTATTGATACCAATCACTAGCATCATCGAAAACAACCTATTAAAATGAAGA

Primer annealing sites marked by: yellow for F1p, green for F2p, cyan for F3p, magenta for F4p, red for F5p, olive for R1p, dark grey for R3p, double underline for R4p, brown-green for R6p

Section S2. Characterization of primer pairs with real-time PCR

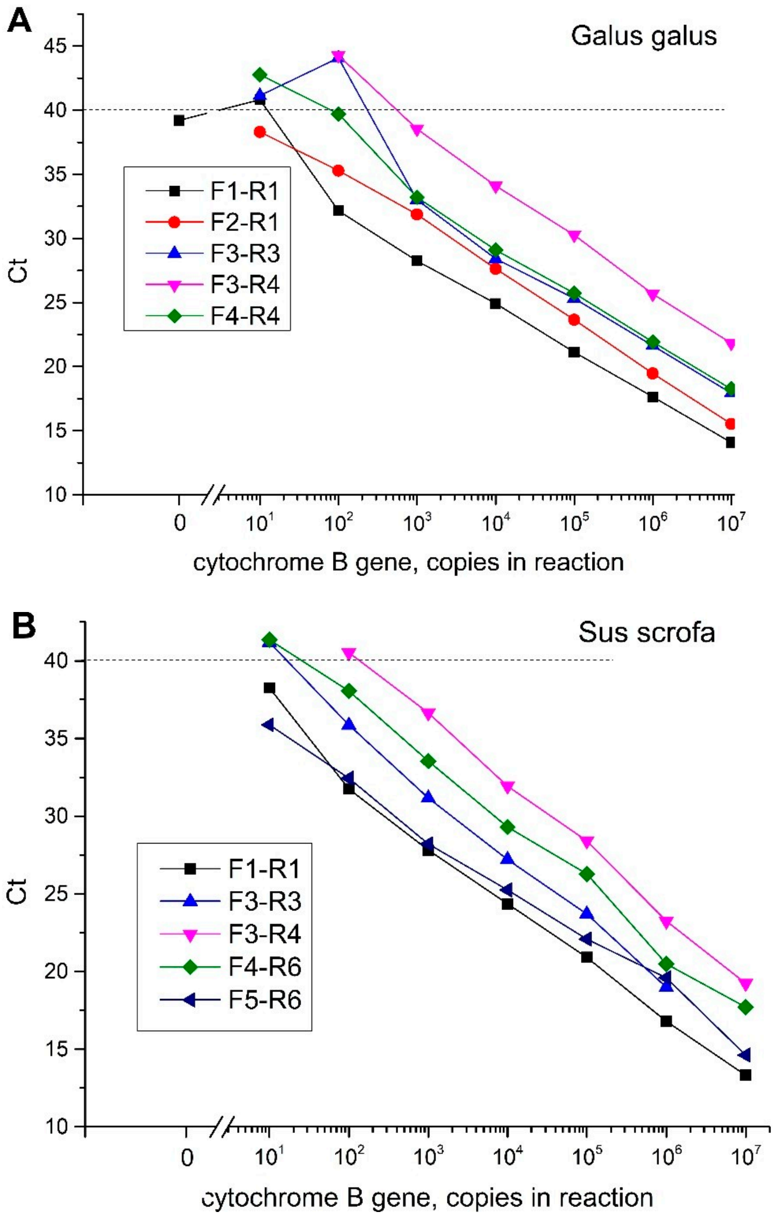

Figure S1. Sensitivity of qPCR with different primers for detection of purified cytB gene of chicken (A) and pig (B).

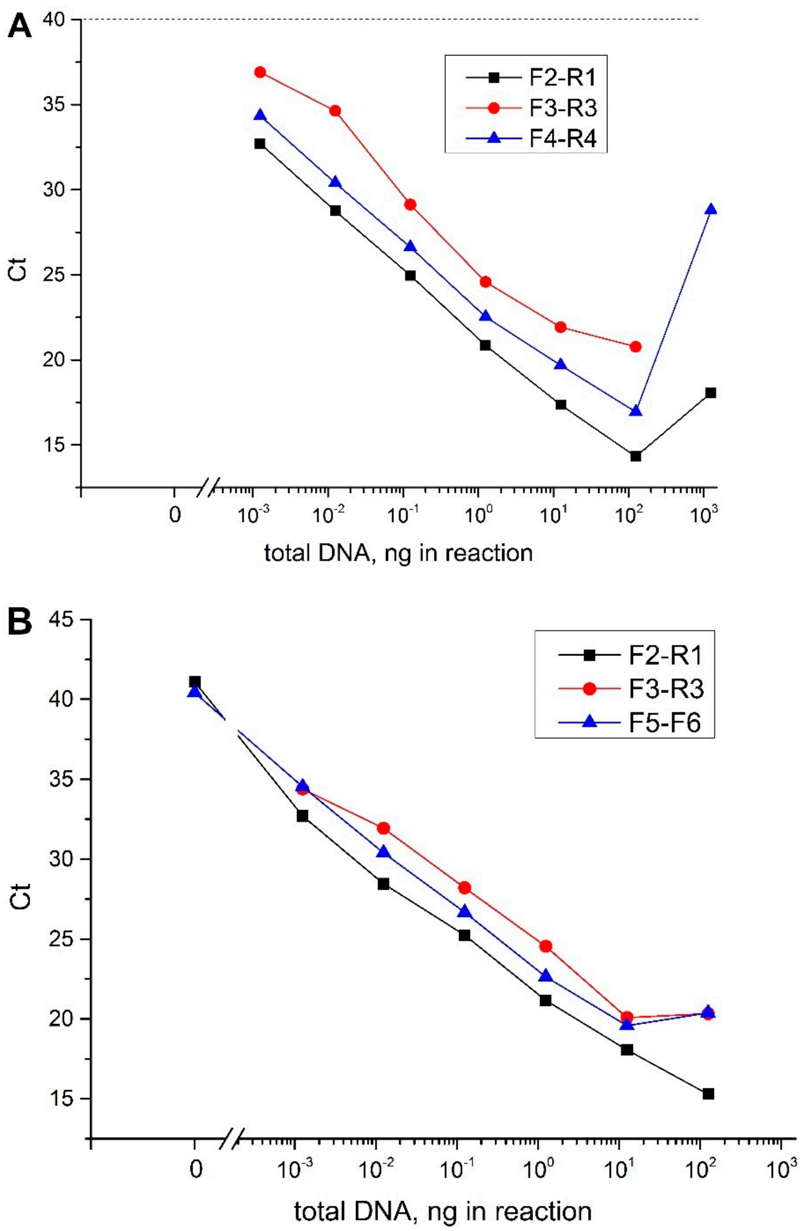

Figure S2. Sensitivity of qPCR with different primers for detection of total DNA extracted from chicken (A) and pig (B) meat.

Section S3. Verification of the developed test with meat samples

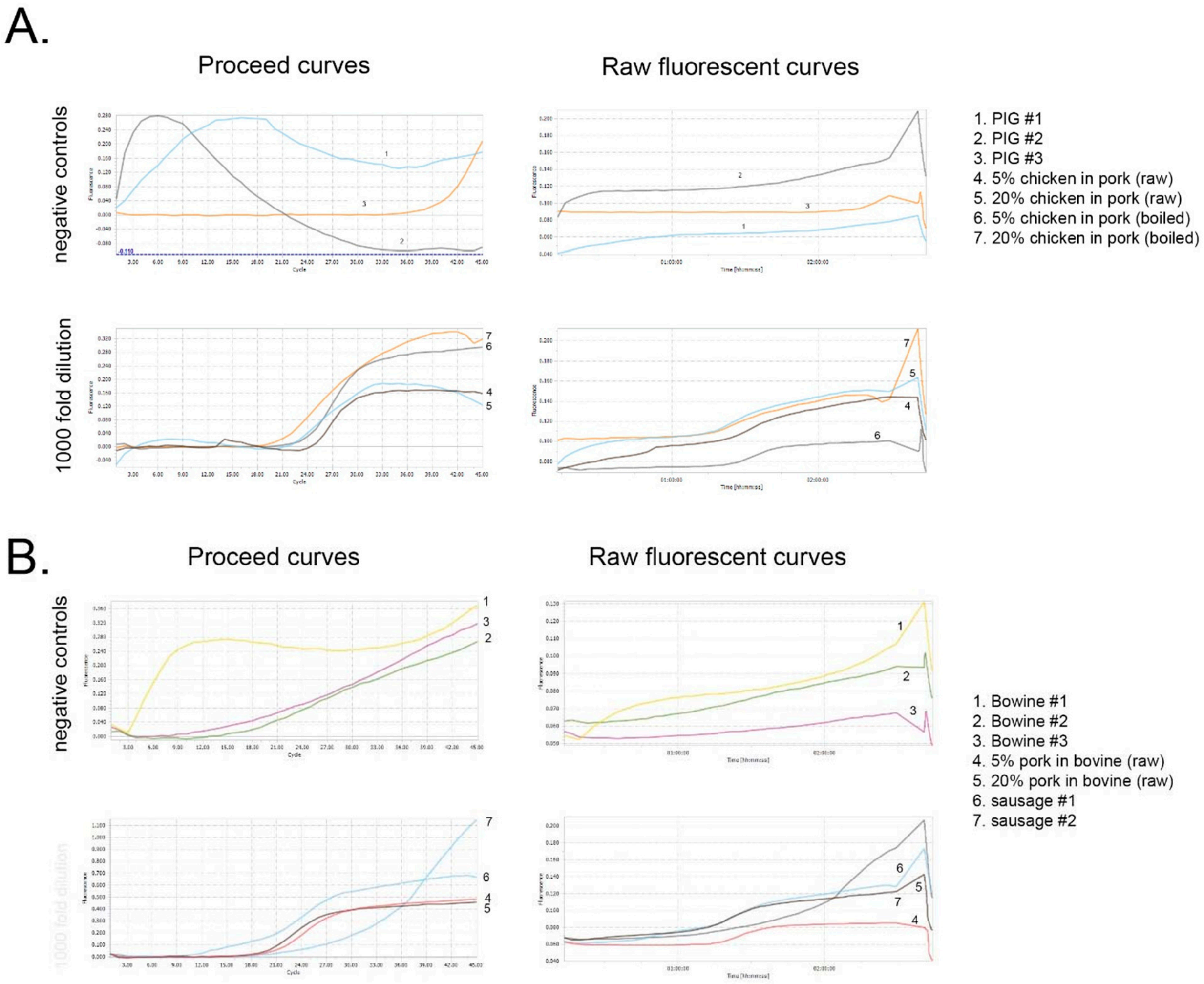

Figure S3. Detection of chicken (A) or pig (B) DNA in meat samples (after accurate DNA extraction) by qPCR.

Section S4. Synthesis of gold nanoparticles

We added 1 mL of 1% $HAuCl_4$ to 95 mL of deionized water and heated to boiling point, then 4 mL of 1% sodium citrate was added while the mixture was stirred. The mixture was boiled for 25 min then cooled and stored at 4°C.

The size and shape of the synthesized GNP were analyzed by dynamic laser scattering (DLS) and imaging with transmission electron microscopy (TEM). DLS was performed by ZetaSizer Nano (Malvern, USA) and showed average Z-size of the GNPs as 20 nm. TEM was performed by JEM CX-100 electron microscope (Jeol, Japan) and confirmed the size of the GNPs. Concentration of GNPs was estimated by optical absorption at 520 nm with Lybra Biochrome spectrometer (USA).
